# Childhood cancer incidence in a cohort of twin babies

**DOI:** 10.1054/bjoc.2001.1799

**Published:** 2001-06

**Authors:** M F G Murphy, D Whiteman, K Hey, M Griffith, L Gill, M J Goldacre, T J Vincent, K Bunch

**Affiliations:** Imperial Cancer Research Fund, General Practice Research Group, Institute of Health Sciences, Old Road, Headington, Oxford OX3 7LF, UK; Unit of Health-Care Epidemiology, University of Oxford, Institute of Health Sciences, Old Road, Headington, Oxford OX3 7LF, UK; Childhood Cancer Research Group, University of Oxford, 57 Woodstock Road, Oxford OX2 6HJ, UK

**Keywords:** twins, childhood cancer, childhood mortality

## Abstract

We studied childhood cancer incidence in a population-based twin cohort using record linkage to the National Registry of Childhood Tumours. After correcting for mortality, an incidence deficit was observed (Standardized Incidence Ratio (SIR) 79; 95% Confidence Interval (CI) 39–120). Pooled analysis with data from published cohort studies indicates a similar significant incidence reduction (SIR 81, 95% CI 67–96). Further studies are warranted. © 2001 Cancer Research Campaign http://www.bjcancer.com


*British Journal of Cancer* (2001) **84**(11), 1460–1462
© 2001 Cancer Research Campaign
doi: 10.1054/ bjoc.2001.1799, available online at http://www.idealibrary.com on IDEAL®

http://www.bjcancer.com

Short Communication

### Childhood cancer incidence in a cohort of twin babies

**MFG Murphy[1], D Whiteman[1], K Hey[1], M Griffith[2], L Gill[2], MJ Goldacre[2], TJ Vincent[3] and K Bunch[3]**

[1]Imperial Cancer Research Fund, General Practice Research Group, Institute of Health Sciences, Old Road, Headington, Oxford OX3 7LF, UK; [2]Unit of Health-Care Epidemiology, University of Oxford, Institute of Health Sciences, Old Road, Headington, Oxford OX3 7LF, UK; [3]Childhood Cancer Research Group, University of Oxford, 57 Woodstock Road, Oxford OX2 6HJ, UK

**Summary** We studied childhood cancer incidence in a population-based twin cohort using record linkage to the National Registry of Childhood Tumours. After correcting for mortality, an incidence deficit was observed (Standardized Incidence Ratio (SIR) 79; 95% Confidence Interval (CI) 39–120). Pooled analysis with data from published cohort studies indicates a similar significant incidence reduction (SIR 81, 95% CI 67–96). Further studies are warranted. © 2001 Cancer Research Campaign http://www.bjcancer.com

**Keywords**: twins; childhood cancer; childhood mortality

Several studies have suggested that twins develop fewer childhood cancers than singletons (Hewitt et al, 1966; Jackson et al, 1969; Norris and Jackson, 1970; Windham et al, 1985; Inskip et al, 1991; Rodvall et al, 1992). Some lacked detailed information on mortality or migration and approximated the estimate of twins at risk, generating concern that this unexpected cancer deficit might arise from inaccuracies in these estimates. We report here follow-up of an unselected population-based cohort of twins born in Southern England for whom complete records of childhood death and cancer incidence were available.

### METHODS

The Oxford Record Linkage Study (ORLS) is assembled from computerized abstracts of hospital inpatient and day-case records, birth registration details and birth certificates of all babies born in the area (Acheson, 1967). The ORLS area covered Oxford City for 1963, Oxfordshire for 1964, Oxfordshire and West Berkshire from 1965 to 1974 and Oxfordshire, Berkshire, Buckinghamshire and Northamptonshire from 1975 onwards. Birthweight was available from clinical data about babies born in Oxfordshire and West Berkshire and from data made available to the Office of Population Censuses and Surveys (OPCS), now the Office for National Statistics (ONS), from birth notifications at the time of birth registration. Death certificates were available for all residents of the above area in England and Wales and additionally until 1992 for those born in the study area who died under age 15 irrespective of their place of residence in England and Wales.

Using name, National Health Service Central Register (NHSCR) number, sex and birth date as identification, we record-linked twins born 1963–1989 to the National Registry of Childhood Tumours (NRCT) maintained by the Childhood Cancer Research Group (CCRG) in Oxford to identify twins with cancer. The NRCT includes information on children resident in England, Wales or Scotland and aged under 15 years at the time of diagnosis with a malignant neoplasm at any site or any brain tumour. The principal sources of ascertainment are the National Cancer Registration schemes which cover the whole of Britain through a network of regional registries, the register of children under the care of members of the United Kingdom Children's Cancer Study Group (UKCCSG), local population-based childhood cancer registries in several regions, death certificates and entrants to the Medical Research Council (MRC) leukaemia trials. At the time of the present analysis the NRCT was complete up to 1995. It is the best centralized source of data on childhood cancer in Britain (Stiller et al, 1995).

For comparison with cancers observed, expected numbers were calculated for sex, age group (0–4, 5–9 and 10–14 years) and 12 diagnostic groups of childhood malignancy by applying NRCT incidence rates for Britain to the live twin births. We corrected only for infant mortality because there were so few older deaths. Few details of emigration were known, so we estimated the maximum number of twins who had emigrated by record linking the cohort to the NHSCR to identify those definitely alive and in this country in 1991 (Gill et al, 1993).

We systematically reviewed the published literature to identify studies providing risk estimates of childhood cancer incidence or mortality in twins. Results for incidence and mortality were separately pooled using a fixed effects model and weighting the result for each study by the inverse of its variance.

### RESULTS

We identified 13 009 twins born 1963–1989 on the ORLS files and believe the cohort to be almost complete. Linkage to NHSCR suggested that emigration would have affected expected values by at most 5%, and probably considerably less.

Fifteen definite childhood malignancies were registered before 31 December 1995. Thirteen twin pairs were discordant for cancer, and one pair concordant for lymphoid leukaemia. Their birth order distribution was unremarkable. Three were three astrocytomas in females and three leukaemias in males. Table 2 shows that by age 15 about 18.9 cancers were expected compared to 15 observed producing a non significant standardized incidence ratio of 79 with

*Received 12 September 2000*
*Revised 26 February 2001*
*Accepted 28 February 2001*

*Correspondence to:* M Murphy

**Table 1** Characteristics of the registered childhood cancers amongst twins born 1963–89 with diagnoses in the period 1963–95

| Age at diagnosis (years) | Sex | Diagnosis | Sex combination of twin pair | Birthweight (g) | Birth order |
|---|---|---|---|---|---|
| 0 | M | Soft tissue sarcoma | MM | – | 2 |
| 4 | M | Wilms' tumour (renal) | MM | – | 1 |
| 6 | F | Astrocytoma | FF | – | 2 |
| 0 | M | Wilms' tumour (renal) | MM | 2050 | 1 |
| 4 | M | Unspecified lymphoma | MF | 3030 | 1 |
| 7 | F | PNET (medulloblastoma) | FM | – | 1 |
| 6 | M | Ewing's sarcoma (bone) | MM | – | 2 |
| 0 | M | Lymphoid leukaemia* | MM | 1200 | 2 |
| 0 | M | Lymphoid leukaemia* | MM | 1720 | 1 |
| 4 | M | Non-Hodgkin lymphoma | MM | 3289 | 2 |
| 1 | F | Neuroblastoma | FF | 2690 | 2 |
| 3 | M | Lymphoid leukaemia | MF | 3430 | 1 |
| 10 | F | Astrocytoma | FF | 2750 | 2 |
| 5 | F | Astrocytoma | FF | 3420 | 2 |
| 12 | M | Burkitt's lymphoma | MM | 2220 | 2 |

*Probable concordant monozygotic twin pair.

a 95% Confidence Interval (CI) of 39–120. The concentration of observed and expected numbers at lower ages is explained by the age distribution of childhood cancer and by the fact that more recent birth cohorts had not yet reached the older age groups by 1995. For both sexes combined there were fewer cancers than expected in every age group. No value observed in any age group differed significantly from that expected. No important differences were observed in the ratio of observed to expected cancers according to the sex combination of twin pairs in the cohort (12 versus 12.83 in like-sex pairs and three versus 5.88 in unlike-sex pairs).

Birthweight was available for only 9022 babies in the cohort, largely because administrative procedures varied over time. Bias is therefore unlikely but the birthweight of twins is considerably lower on average than for singletons. Examination of observed versus expected cancers by birthweight groups indicated non-significant incidence deficits below 3000 g (and among the group with unknown birthweight) and an excess in those weighing 3000 g or above (data not shown).

**Table 2** Observed and expected total childhood cancer incidence by (a) sex and age at diagnosis and (b) sex combination of twin pairs in the cohort

| (a) | | Age (years) at diagnosis | | | |
|---|---|---|---|---|---|
| | | 0–4 | 5–9 | 10–14 | Total |
| Observed | M | 8 | 1 | 1 | 10 |
| | F | 1 | 3 | 1 | 5 |
| | M + F | 9 | 4 | 2 | 15 |
| Expected | M | 5.35 | 2.97 | 2.02 | 10.34 |
| | F | 4.56 | 2.32 | 1.63 | 8.51 |
| | M + F | 9.91 | 5.29 | 3.66 | 18.86 |

| (b) Sex combination of twin pairs* | | | |
|---|---|---|---|
| Expected | | Observed | |
| Males in MM | 8 | Males in MM | 7.11 |
| Males in MF | 2 | Males in MF | 3.18 |
| Females in FF | 4 | Females in FF | 5.72 |
| Females in MF | 1 | Females in MF | 2.70 |

*Information on sex combination was missing for about 1% of the twins and they are excluded from this analysis.

Table 3 shows the results of the five previous prospective studies of cancer occurrence in twins (and our own). Of six heavily overlapping studies of childhood cancer from Sweden, (Forsberg and Källen, 1990; Zack et al, 1991; Cnattingius et al, 1995; Linet et al, 1996; Mogren et al, 1999; Rodvall et al, 1992), only one (Rodvall et al, 1992) reported a numerical risk for all childhood cancers in twins, that could therefore be included in a pooled analysis. Only one retrospective case-control study was identified (Savitz and Ananth, 1994) reporting a non-significantly increased odds ratio 1.5 (0.4–5.0). Its inclusion does not affect the results of pooled analysis and we have excluded it because of its design. Fewer cancers were observed than expected in every study, whether considering incidence or mortality and in almost every case when subdividing available data into the leukaemias and all other cancers. Pooled analysis of the four studies presenting incidence data produced a significantly low total cancer standardized incidence ratio of 81 with 95% CI (67–96). Pooling of the three mortality studies also produced a significantly low standardized mortality ratio for all cancers of 85 with 95% CI (74–95). In neither instance was there any evidence of significant heterogeneity.

### DISCUSSION

ORLS twins experienced some 20% fewer childhood cancers than expected. Our cohort had the advantages of lack of selection, a population base, and information on individual mortality and aggregate migration. By linkage to the NRCT with excellent identifiers both observed and expected numbers of incident cancers, though small, are likely to be accurate. In our study the higher still birth rate and infant mortality of twins was also seen in the first few years of childhood, as described by others (e.g. Rodvall et al, 1992) though at much lower absolute rates than in infancy.

The ORLS deficit of twin childhood cancer agrees with the findings of other cohort studies (Hewitt et al, 1966; Jackson et al, 1969; Norris and Jackson, 1970; Windham et al, 1985; Inskip et al, 1991; Rodvall et al, 1992). Our study is most comparable to that of Windham et al in terms of size and the period of twin birth (Windham et al, 1985). Both cohorts would have been less exposed to prenatal X-rays than the older studies, because of alternative obstetric methods of confirming twins, assessing fetal

**Table 3** Cancer occurrence in twins versus single births or the general population of children

| | United Kingdom (Hewitt et al, 1966) | California (Jackson et al, 1969; Norris and Jackson, 1970 | Norway (Windham et al, 1985) | Connecticut (Inskip et al, 1991) | Sweden (Rodvall et al, 1992) | | ORLS (Present study) |
|---|---|---|---|---|---|---|---|
| Study outcome | Mortality | Mortality | Incidence | Incidence | Incidence | Mortality | Incidence |
| Year of birth | 1943–63 | 1940–64 | 1967–79 | 1930–69 | 1952–67 | | 1963–89 |
| Cancer (O/E) | | | | | | | |
| Total cancer | (121/152)[a] | (100/111) | (14/15.6)[b] | (31/46.4) | 59/61.7 | 41/45.6 | 15/18.9 |
| Leukaemia | – | (48/52) | (4/5.7)[b] | (13/15.6) | 17/18.1 | 19/21.6 | 3/6.4 |
| All other cancers | – | (52/59.3) | (10/9.9)[b] | (18/30.8) | 42/43.6 | 22/24 | 12/12.5 |

[a]Hewitt et al adjusted the expected number of cancers upward to account for twins' greater frequency of exposure to prenatal X-rays. [b]Expected number of cancers estimated from relative risk and observed number of cancers for Windham et al. O = observed; E = expected. Modified from Inskip et al, 1991 and Murphy, 1995.

growth and imaging the fetal position. A review of previous studies commented that although there were no significant differences in cancer incidence between twins and singletons in any one of the studies, the aggregate data (including their own) pointed to a slightly lower risk amongst twins (Inskip et al, 1991). Pooling the data reviewed by Inskip et al (Inskip et al, 1991), the subsequent data of Rodvall et al (Rodvall et al, 1992) and our own indicates a significant deficit of 15–20% in incident and fatal cancers of all types. Only the Connecticut study (Inskip et al, 1991) reported childhood cancer incidence by sex of the co-twin, though another study (Swerdlow et al, 1996) reported detailed data for childhood and young adulthood combined. As here, neither found any strong evidence that risk of all cancers was influenced by sex combination of the twin pair, or birth order.

No prospective study has found an overall excess, despite differences in study size, design, population and outcome and it seems difficult to conclude that this consistent finding of a deficit could be due to methodological artefact. The tendency to lower birthweight of twins (perhaps because of fewer cell divisions or altered growth factor exposure) and the selection effects of high stillbirth and death rates in infancy may be relevant (Forsberg and Kallen, 1990; McKinney et al, 1999; Ross et al, 1996; Roman et al, 1997). Our birthweight data provide some slight support for the former hypothesis. Another possibility may be prenatal loss of potentially concordant pairs (Hewitt et al, 1966). There is increasing recognition that the scale on which twins 'vanish' and are delivered as a singleton pregnancy may be considerable. Further studies incorporating additional clinical information, may explain the emerging finding of decreased childhood cancer incidence in twins.

### ACKNOWLEDGEMENTS

We would like to thank Kate Bowe of the ICRF General Practice Research Group for typing the manuscript. The Unit of Health-Care Epidemiology receives support from the South East Regional Office of the NHS Executive. The Childhood Cancer Research Group is supported by the Department of Health for England and Wales and the Scottish Ministers. The views expressed are not necessarily those of the Department of Health, the Scottish Ministers or ONS. Grant number M3/95 from Wellbeing/Royal College of Obstetricians and Gynaecologists helped to support the final stages of this study.

### REFERENCES

Acheson ED (1967) *Medical record linkage*. Oxford University Press: Oxford

Cnattingius S, Zack MM, Ekbom A, Gunnarskog J, Kreuger A, Linet M and Adami H-O (1995) Prenatal and neonatal risk factors for childhood lymphatic leukaemia. *J Natl Cancer Inst* **87**: 908–914

Forsberg JG and Källen B (1990) Pregnancy and delivery characteristics of women whose infants develop child cancer. *APMIS* **98**: 37–42

Gill LE, Goldacre M, Simmons H, Bettley G and Griffith M (1993) Computerised linking of medical records: methodological guidelines. *J Epidemiol Community Health* **47**: 316–319

Hewitt D, Lashof JC and Stewart AM (1966) Childhood cancer in twins. *Cancer* **19**: 157–161

Inskip PD, Harvey EB, Boice JD, Stone BJ, Matanoski G, Flannery JT and Fraumeni JF Jr (1991) Incidence of childhood cancer in twins. *Cancer Causes Control* **2**: 315–324

Jackson EW, Norris FD and Klauber MR (1969) Childhood leukaemia in California born twins. *Cancer* **23**: 913–920

Linet MS, Gridley G, Cnattingius S, Nicholson HS, Martinsson U, Glimelius B, Adami H-O and Zack M (1996) Maternal and perinatal risk factors for childhood brain tumours (Sweden). *Cancer Causes Control* **7**: 437–448

McKinney PA, Juszczak E, Findlay E, Smith K and Thomson CS (1999) Pre- and perinatal risk factors for childhood leukaemia and other malignancies: a Scottish case-control study. *Br J Cancer* **80**: 1844–1851

Mogren I, Damber L, Tavelin B and Högberg U (1999) Characteristics of pregnancy and birth and malignancy in the offspring (Sweden). *Cancer Causes Control* **10**: 85–94

Murphy MFG (1995) The association of twinning with long term disease. In: *Multiple pregnancy*, Ward RH and Whittle M (eds) pp 14–29 RCOG: London

Norris FD and Jackson EW (1970) Childhood cancer deaths in California born twins. A further report on types of cancer found. *Cancer* **25**: 212–218

Rodvall Y, Hrubec Z, Pershagen G, Ahlbom A, Bjurman A and Boice JD Jr (1992) Childhood cancer amongst Swedish twins. *Cancer Causes Control* **3**: 527–532

Roman E, Ansell P and Bull D (1997) Leukaemia and non-Hodgkin's lymphoma in children and young adults: are prenatal and neonatal factors important determinants of disease. *Br J Cancer* **76**: 406–415

Ross JA, Perentesis JP, Robison LL and Davies SM (1996) Big babies and infant leukaemia: a role for insulin-like growth factor-1? *Cancer Causes Control* **7**: 553–559

Savitz DA and Ananth CV (1994) Birth characteristics of childhood cancer cases, controls and their siblings. *Pediatr Hematol Oncol* **11**: 587–599

Stiller CA, Allen MB and Eatock EM (1995) Childhood cancer in Britain: The national registry of childhood tumours and incidence rates 1978–87. *Eur J Cancer* **31A**: 2028–2034

Swerdlow AJ, De Stavola B, Maconochie N and Siskind V (1996) A population based study of cancer risk in twins: relationships to birth order and sexes of the twin pair. *Int J Cancer* **67**: 472–478

Windham GC, Bjerkedal T and Langmark F (1985) A population-based study of cancer incidence in twins and in children with congenital malformations or low birthweight, Norway 1967–80. *Am J Epidemiol* **121**: 49–56

Zack M, Adami H-O and Ericson A (1991) Maternal and perinatal risk factors for childhood leukaemia. *Cancer Res* **51**: 3696–3701

© 2001 Cancer Research Campaign

